# Quantifying the attributable burden of economic inequality on under-five mortality across ten African countries: a WHO HEAT-based analysis

**DOI:** 10.1186/s12889-025-26002-y

**Published:** 2025-12-17

**Authors:** Alpha Umaru Bai-Sesay, Edward Ellie, Rosetta Doreen Jones, Mohamed S. Bah, Chizaram Anselm Onyeaghala

**Affiliations:** 1 Ministry of Health, National Public Health Agency, Freetown, Sierra Leone; 2 Research and Scientific Division, Sustainable Health Systems, Freetown, Sierra Leone; 3 Insights for Development Impact, Freetown, Sierra Leone; 4 34 Military Hospital Wilberforce, Freetown, Sierra Leone; 5 Department of Internal Medicine, University of Port Harcourt Teaching Hospital, Port Harcourt, Nigeria

**Keywords:** Africa, Under-five mortality, Economic inequality, Health equity, Precision public health, Socioeconomic disparities, WHO HEAT, Sustainable development goals

## Abstract

**Introduction:**

Despite global declines in under-five mortality, African countries continue to experience high rates, with socioeconomic disparities undermining progress toward Sustainable Development Goal 3.2. While wealth gradients in child survival are well documented, the proportion of deaths directly attributable to inequality remains poorly quantified across national contexts.

**Methods:**

We conducted a cross-national secondary analysis using the WHO Health Equity Assessment Toolkit (HEAT) v.6.0. Data from ten African countries for the 2022 reference year were included, comprising a total study population of over 6.5 million live births. Under-five mortality rates were obtained from United Nations Inter-agency Group for Child Mortality Estimation (UN IGME) datasets, disaggregated by wealth quintile and sex. Inequality was assessed using Difference, Ratio, Population Attributable Risk, Population Attributable Fraction, Slope Index of Inequality, and Relative Index of Inequality.

**Results:**

National under-five mortality ranged from 38.0 per 1,000 live births in Rwanda to 117.3 in Niger, with all countries exceeding the SDG target of ≤ 25. Wealth gradients were present in all settings: the absolute difference between poorest and richest quintiles ranged from 11.3 (South Sudan) to 67.0 (Mali). Inequality-attributable burdens were highest in Mali (PAR − 41.9; PAF − 44.7%), Togo (PAR − 25.6; PAF − 42.5%), and Madagascar (PAR − 22.7; PAF − 34.5%). Gradient measures confirmed steep inequities in Mali and Togo (SII ≤ -56.3; RII 2.3). Male children had higher mortality, but sex-attributable fractions were negligible.

**Conclusion:**

Up to 45% of under-five deaths in high-burden African countries are attributable to economic inequality. Embedding equity metrics into child survival strategies and targeting the poorest households are essential to accelerate progress toward ending preventable child deaths. Precision public health targeting the poorest quintiles is crucial to reducing preventable child deaths in Africa.

**Supplementary Information:**

The online version contains supplementary material available at 10.1186/s12889-025-26002-y.

## Introduction

Under-five mortality (U5M) is a sentinel indicator of population health, reflecting both disease burden and the inequities embedded within health systems [1, 2]. Defined as the probability of dying before age five per 1,000 live births, the under-five mortality rate (U5MR) has declined globally by 61% over the past three decades from 94 in 1990 to 37 in 2023, driven by large-scale investments in maternal and child health and the expansion of life-saving interventions [3–5]. Yet these gains remain highly uneven. Sub-Saharan Africa (SSA) bears 58% of all global under-five deaths, on average, for every 1,000 babies born alive, 68 of them will die before reaching age five, nearly fourteen times higher than in high-income countries [2, 6]. Current projections suggest that more than 50 countries, many of them in SSA, are not on track to meet Sustainable Development Goal (SDG) 3.2, which calls for reducing U5MR to ≤ 25 by 2030 [7].

Economic inequality is a central determinant of child survival in Africa [8]. The region’s high-income disparities exemplified by Gini coefficients exceeding 50 in countries such as South Africa, Namibia, and Zambia, translate into unequal access to nutrition, clean water, education, and health care. Children from the poorest households consistently face U5MRs two or more times higher than those from wealthier quintiles [8–10]. Maternal education further compounds these gaps, with children of mothers lacking formal schooling experiencing mortality rates more than double those of peers whose mothers attained higher education [11, 12]. These patterns reinforce intergenerational cycles of disadvantage and slow national progress toward mortality reduction.

The determinants of U5M are well established through the Mosley and Chen framework, which categorizes distal factors, intermediate factors, and proximate factors [13]. Socioeconomic inequities shape exposure to each layer of risk, making wealth disparities among the strongest and most persistent predictors of child survival [14]. Evidence from Demographic and Health Surveys (DHS) and Multiple Indicator Cluster Surveys (MICS) consistently demonstrates that socioeconomic gradients explain a larger share of mortality variation than biological or geographic determinants [15].

Precision public health offers an opportunity to address these inequities by tailoring interventions to the most disadvantaged groups using disaggregated, equity-focused data [16]. The World Health Organization’s Health Equity Assessment Toolkit (HEAT) is central to this paradigm [17, 18]. HEAT harmonizes DHS, MICS, and other sources to generate standardized inequality metrics, such as the Slope Index of Inequality (SII) and Population Attributable Fraction (PAF) that facilitate both within-country and cross-national comparisons [17, 19]. While previous research has described socioeconomic gradients in U5M, the proportion of under-five deaths that could be prevented by eliminating economic inequality remains unquantified and compared systematically across multiple African nations.

This study addresses this gap by applying a cross-national HEAT analysis to ten African countries to estimate the proportion of under-five deaths attributable to economic inequality. Through combining measures of inequality (SII, Relative Index of Inequality [RII]) with measures of attributable burden (Population Attributable Risk [PAR], PAF), we provide actionable estimates of preventable deaths. These findings aim to advance precision public health by integrating equity metrics into policy design and resource allocation, accelerating progress toward SDG 3.2, and contributing to the reduction of inequalities within and among countries as outlined in SDG 10, ensuring that no child’s survival is determined by household wealth.

## Methods

### Study design and setting

We conducted a cross-national, retrospective secondary data analysis to quantify the burden of under-five mortality attributable to economic inequality across ten African countries: Liberia, Madagascar, Malawi, Mali, Mozambique, Niger, Rwanda, South Sudan, Togo, and Uganda. The study period focused on 2022, the most recent year with complete estimates in the World Health Organization’s Health Equity Assessment Toolkit (HEAT, version 6.0; accessed 1 July 2025). Country selection was based primarily on the availability of complete, disaggregated U5M data across household wealth quintiles and sex for the 2022 reference year within the HEAT platform, while also ensuring regional diversity in health system capacity, socioeconomic contexts, and epidemiological profiles across Sub-Saharan Africa.

### Population and sampling

The study population comprised all live births in the selected countries, totaling over 6.5 million births in 2022, as reported in HEAT. No primary sampling was performed; instead, we relied on pre-existing nationally representative data derived from Demographic and Health Surveys, Multiple Indicator Cluster Surveys, national censuses, and vital registration systems harmonized by the UN Inter-agency Group for Child Mortality Estimation (UN IGME). Sample sizes ranged from 54,639 births in Togo to 848,271 in Uganda. Eligibility was restricted to children under five years with mortality data disaggregated by household wealth quintile and sex. Sample size determination and statistical power considerations were inherent to DHS and MICS survey designs, which typically ensure ≥ 80% power at α = 0.05 to detect differences in mortality across wealth strata.

### Data sources and variables

The primary outcome was under-five mortality rate, defined as the probability of dying before age five per 1,000 live births. Estimates were extracted from UN IGME datasets integrated within HEAT. It is important to note that these are not raw survey data but statistically modeled reference estimates for 2022, which integrate and smooth all available data sources (e.g., DHS, MICS, censuses) to produce robust and comparable country-level figures.

The main exposure variable was household wealth quintile, classified from Q1 (poorest) to Q5 (richest). Wealth indices are constructed in DHS/MICS using principal component analysis of household assets, dwelling characteristics, and access to services.

A secondary exposure variable was child sex (male or female), recorded from maternal birth histories. No additional covariates were modeled, reflecting the descriptive and inequality-focused nature of the analysis. Following WHO guidance, wealth and sex were selected as key equity stratifiers given their policy relevance and comparability across countries.

### Data extraction and quality control

Data were extracted directly from HEAT, which harmonizes mortality estimates across DHS, MICS, censuses, and civil registration systems. UN IGME applies Bayesian B-spline smoothing, clustering adjustments, and internal consistency checks to account for recall bias, sampling error, and small-sample fluctuations. To verify reliability, we cross-checked HEAT outputs against country-level DHS/MICS reports and confirmed that U5MR estimates matched within ± 1.0 per 1,000 live births. As HEAT data are anonymized and aggregated, no primary data collection or individual-level linkages were conducted.

### Inequality measures and statistical analysis

Analyses were performed using standardized algorithms embedded in HEAT, based on the WHO Handbook on Health Inequality Monitoring. For wealth quintiles, both simple and complex inequality measures were computed:

Simple measures: Difference (D): absolute difference in U5MR between Q1 and Q5. Ratio (R): relative ratio of U5MR between Q1 and Q5.

Complex measures: Population Attributable Risk: absolute excess mortality (per 1,000 live births) attributable to inequality, comparing the national U5MR to the rate in the richest quintile. Population Attributable Fraction (PAF): proportion (%) of deaths attributable to inequality. Slope Index of Inequality: regression-based measure of the absolute gradient in mortality across all wealth quintiles. Relative Index of Inequality: relative gradient measure accounting for the entire wealth distribution.

For sex, D, R, PAR, and PAF were computed, while SII and RII were not applicable due to the binary nature of the stratifier. All measures were calculated automatically within HEAT, which ensures methodological consistency across settings. HEAT computes 95% confidence intervals for complex measures like SII and RII using a bootstrapping approach, which accounts for the sampling variability of the underlying survey data used in the UN IGME models. Outputs were exported to Microsoft Excel (version 16.76) for formatting, descriptive summaries, and preparation of figures and tables. Statistical significance was assessed at *p* < 0.05, with 95% confidence intervals (CIs) reported where available.

### Ethical considerations

The study relied exclusively on aggregated, anonymized, publicly available data from WHO HEAT repository. No individual-level identifiers were accessed, and no human subjects were directly involved. In accordance with international guidelines for secondary analyses of de-identified data, institutional ethics approval was not required.

## Results

The analysis included ten African countries with complete under-five mortality estimates disaggregated by household wealth quintile and sex for 2022. Together, these countries accounted for over 6.5 million live births, ranging from 54,639 in Togo to 848,271 in Uganda. All countries had valid estimates for five wealth quintiles and both sexes, with no missing strata.

Marked variation was observed across settings (Supplementary Table S1). The highest national U5MRs were recorded in Niger (117.3 per 1,000 live births), South Sudan (98.8), and Mali (93.8). The lowest were observed in Rwanda (38.0), Malawi (40.1), and Uganda (40.5). Intermediate levels were noted in Liberia (73.2), Madagascar (65.8), Mozambique (66.2), and Togo (60.4). All ten countries remained above the SDG 3.2 target of ≤ 25 per 1,000 live births.

A clear wealth gradient was evident in every country (Table 1, see Supplementary Table S1 for detailed mortality rates by quintile). The absolute difference in U5MR between the poorest (Q1) and richest (Q5) quintiles ranged from 11.3 deaths per 1,000 in South Sudan to 67.0 in Mali. Substantial disparities were also observed in Togo (45.4), Niger (40.9), and Madagascar (36.5).

**Table 1:** Under-five mortality rates by wealth quintile and sex across ten African countries, 2022

Country	Dimension	D	PAF (95% CI)	PAR (95% CI)	*R*	RII (95% CI)	SII (95% CI)
Liberia	Economic status (wealth quintile)	17.6	−14.2 (−14.2, −14.2)	−10.4 (−12.8, −8.0)	1.3	0.7 (0.7, 0.8)	−23.9 (−31.7, −16.1)
	Sex	−11.7	0.0 (−0.0, 0.0)	0.0 (−1.2, 1.2)	0.9	–	–
Madagascar	Economic status (wealth quintile)	36.5	−34.5 (−34.5, −34.4)	−22.7 (−23.5, −21.8)	1.8	0.5 (0.4, 0.7)	−45.0 (−66.0, −24.0)
	Sex	−10.6	0.0 (−0.0, 0.0)	0.0 (−0.5, 0.5)	0.9	–	–
Malawi	Economic status (wealth quintile)	16.6	−24.0 (−24.1, −24.0)	−9.6 (−10.5, −8.8)	1.5	0.6 (0.5, 0.7)	−21.3 (−28.2, −14.4)
	Sex	−9	0.0 (−0.0, 0.0)	0.0 (−0.5, 0.5)	0.8	–	–
Mali	Economic status (wealth quintile)	67	−44.7 (−44.7, −44.6)	−41.9 (−42.8, −40.9)	2.3	0.4 (0.3, 0.7)	−80.6 (−122.4, −38.7)
	Sex	−10.2	0.0 (−0.0, 0.0)	0.0 (−0.6, 0.6)	0.9	–	–
Mozambique	Economic status (wealth quintile)	27.3	−23.5 (−23.5, −23.4)	−15.5 (−16.4, −14.7)	1.5	0.6 (0.5, 0.7)	−34.1 (−43.5, −24.6)
	Sex	−8.9	0.0 (−0.0, 0.0)	0.0 (−0.4, 0.4)	0.9	–	–
Niger	Economic status (wealth quintile)	40.9	−29.5 (−29.5, −29.5)	−34.6 (−35.6, −33.5)	1.5	0.6 (0.4, 1.0)	−55.7 (−113.2, 1.8)
	Sex	−6.7	0.0 (−0.0, 0.0)	0.0 (−0.6, 0.6)	0.9	–	–
Rwanda	Economic status (wealth quintile)	20.1	−26.6 (−26.6, −26.6)	−10.1 (−11.2, −9.1)	1.7	0.5 (0.5, 0.6)	−24.0 (−27.6, −20.3)
	Sex	−6.8	0.0 (−0.0, 0.0)	0.0 (−0.6, 0.6)	0.8	–	–
South Sudan	Economic status (wealth quintile)	11.3	−3.9 (−3.9, −3.9)	−3.9 (−5.9, −1.8)	1.1	0.8 (0.7, 1.0)	−22.2 (−39.8, −4.6)
	Sex	−9.8	0.0 (−0.0, 0.0)	0.0 (−1.0, 1.0)	0.9	–	–
Togo	Economic status (wealth quintile)	45.4	−42.5 (−42.5, −42.4)	−25.6 (−27.1, −24.2)	2.3	0.4 (0.3, 0.5)	−56.3 (−75.2, −37.5)
	Sex	−9.8	0.0 (−0.0, 0.0)	0.0 (−0.9, 0.9)	0.8	–	–
Uganda	Economic status (wealth quintile)	21.8	−28.5 (−28.5, −28.5)	−11.5 (−12.1, −11.0)	1.8	0.5 (0.4, 0.7)	−25.1 (−31.9, −18.3)
	Sex	−8.6	0.0 (0.0, 0.0)	0.0 (−0.3, 0.3)	0.8	–	–

**Fig. 1:**
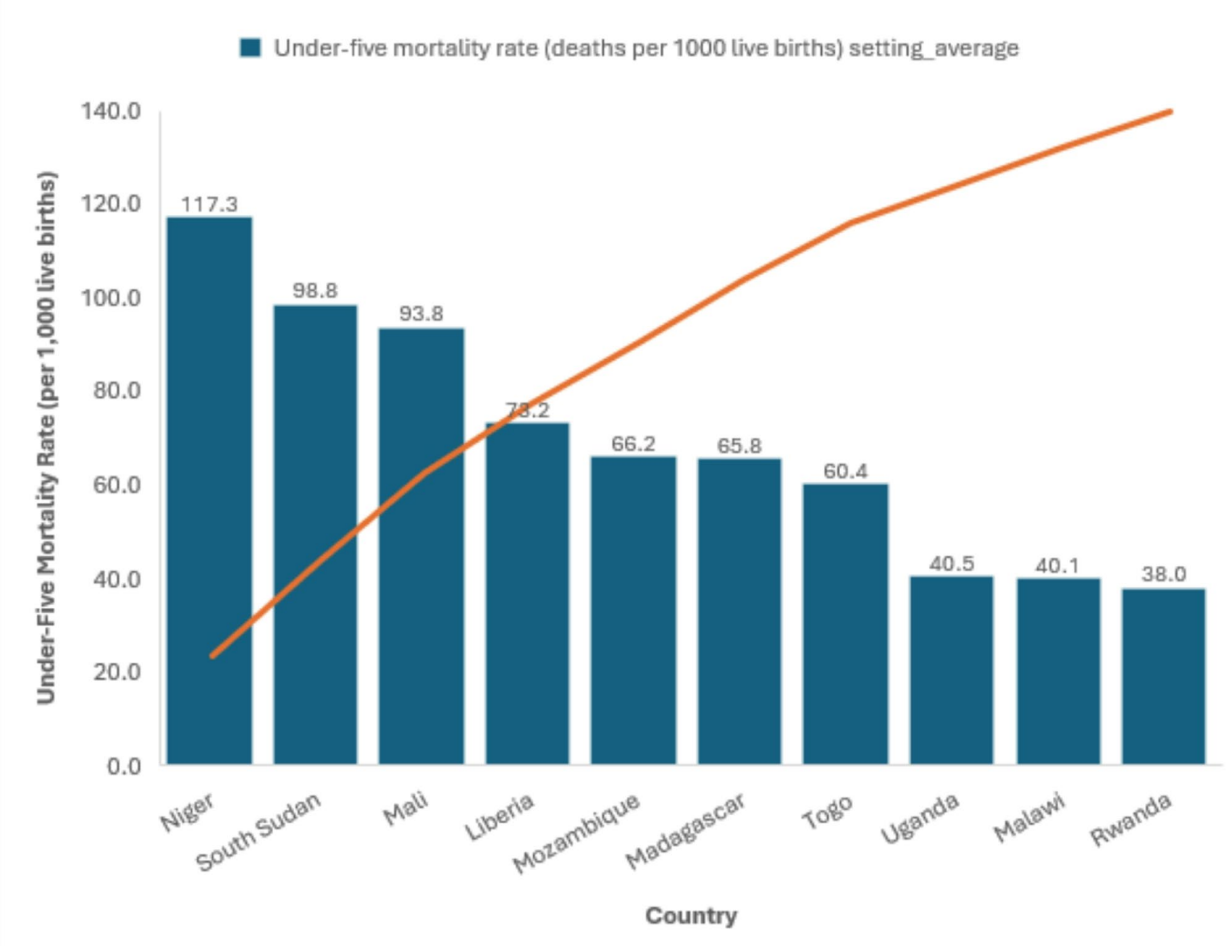
Under-five mortality rates (per 1,000 live births) across ten African Countries

The burden of under-five mortality attributable to economic inequality was substantial (Fig. 1). Measures of attributable burden confirmed the magnitude of inequality. Mali had the highest Population Attributable Risk (PAR = −41.9 deaths per 1,000), followed by Niger (−34.6), Togo (−25.6), and Madagascar (−22.7). These values represent the number of deaths per 1,000 live births that could be prevented if all children had the U5MR of the richest quintile.

Relative burdens were also striking. Population Attributable Fraction values reached − 44.7% in Mali, −42.5% in Togo, and − 34.5% in Madagascar, indicating that one-third to nearly half of all under-five deaths in these countries were attributable to economic inequality.

Gradient-based measures reinforced these findings. The Slope Index of Inequality was steepest in Mali (−80.6), Togo (−56.3), and Niger (−55.7), while Relative Index of Inequality was highest in Mali (2.3) and Togo (2.3), signifying that children in the poorest quintiles faced more than double the mortality risk of their wealthiest peers. By contrast, South Sudan exhibited a modest absolute gap (PAR = −3.9) and a Relative Index of Inequality of 0.8 (95% CI: 0.7, 1.0). This RII value, which is less than 1 and whose confidence interval includes 1, indicates a less steep and non-significant relative socioeconomic gradient compared to other countries, consistent with the observed pattern where the mortality rate in the richest quintile was also exceptionally high and close to that of the poorest.

Male children consistently experienced higher mortality than females across all countries (Table 1). The sex-based absolute difference ranged from − 6.7 in Niger to −11.7 in Liberia. Ratios ranged from 0.8 to 0.9, confirming a modest but consistent male disadvantage. However, attributable measures (PAR and PAF) were uniformly 0.0, reflecting the binary structure of the stratifier and the absence of a definable reference group.

When ranked by PAR, the countries with the largest inequality-driven absolute burdens were Mali (−41.9), Niger (−34.6), and Madagascar (−22.7). When ranked by PAF, the leading countries were Mali (−44.7%), Togo (−42.5%), and Madagascar (−34.5%). These rankings were consistent across inequality measures, underscoring the urgent need for equity-sensitive interventions in these settings.

## Discussion

This cross-national analysis quantified the absolute and relative burden of under-five mortality attributable to economic inequality in ten African countries using standardized measures from the WHO Health Equity Assessment Toolkit. The results show that socioeconomic disparities in child survival remain pervasive and structurally embedded across diverse contexts. In Mali, Madagascar, and Togo, up to 45% of under-five deaths were attributable to inequality, with Population Attributable Risks exceeding 20 deaths per 1,000 live births. The Slope Index of Inequality and Relative Index of Inequality confirmed steep mortality gradients, underscoring the disproportionate burden borne by the poorest quintiles. While male children consistently experienced higher U5M, sex-based Population Attributable Fractions were negligible, highlighting the dominant role of economic disparities over biological vulnerability. Collectively, these findings reveal that inequality is not a marginal modifier but a central driver of preventable child mortality.

The persistence of large wealth-based gradients in U5M aligns with longstanding evidence from Demographic and Health Surveys and Multiple Indicator Cluster Surveys, which consistently document mortality risks 1.5 to 3 times higher among the poorest quintiles compared to the wealthiest [20, 21]. However, this study makes three novel contributions.

Through applying HEAT’s standardized methodology across multiple countries, it enables direct cross-national comparisons. Most previous studies have often been country-specific, limited to descriptive gradients, or employed non-comparable methods. Here, the distinction between absolute burden (PAR) and relative burden (PAF) proved particularly revealing. For example, Niger had the highest national U5MR (117.3 per 1,000), yet Mali exhibited the largest inequality-driven burden (PAR − 41.9; PAF − 44.7%). This distinction suggests that while Niger’s child mortality crisis is more severe in absolute terms, Mali’s mortality pattern is more profoundly shaped by inequity. Such insights are crucial for policy targeting and efficient allocation of resources.

The use of gradient-based measures (SII and RII) adds interpretive depth. Mali’s steep SII (−80.6) indicates a wide absolute gap across the wealth spectrum, while its RII (2.3) reflects a mortality risk more than twice as high among the poorest relative to the average child. While South Sudan’s modest PAR (−3.9) but elevated RII (0.8) illustrates how relative disadvantage persists even when the absolute burden is lower. These findings highlight the importance of interpreting multiple inequality measures in tandem, avoiding simplistic reliance on a single indicator. The stark inequality-driven burdens in Mali, Togo, and Madagascar warrant contextual consideration. These countries face intersecting challenges that likely exacerbate wealth-based disparities in child survival. Mali and South Sudan have experienced prolonged political instability and conflict, which disrupt health systems and disproportionately affect the poor. Togo and Madagascar contend with significant governance challenges and high levels of poverty, which can limit the reach and quality of public health services to the most marginalized communities. Furthermore, all three countries have fragile health systems with documented barriers to access, including financial hardship and geographic inaccessibility, which are most severe for the poorest quintiles. These factors create a perfect storm where economic inequality translates directly into vastly unequal survival chances for children.

Additionally, the study demonstrates the value of extending HEAT from descriptive monitoring to quantification of preventable deaths. The shift from reporting disparities to estimating attributable fractions reframes inequality as a modifiable risk factor, positioning precision public health as a tool for identifying and reducing avoidable child deaths [17, 19]. This approach resonates with recent calls to embed health equity metrics into the design of interventions and global financing frameworks [22, 23].

The modest but consistent male disadvantage in U5M, with absolute differences of 6–12 deaths per 1,000, aligns with biological evidence of greater vulnerability to neonatal complications and infections among boys [24, 25]. However, the negligible PAFs for sex emphasize that biological differences pale in comparison to structural inequities in determining survival. This highlights the need to focus on social determinants as the dominant leverage points for reducing mortality.

### Policy and programmatic implications

The evidence presented has several critical implications for health policy and programming. Countries such as Mali, Madagascar, Togo, and Niger face the heaviest inequality-driven burdens and therefore need to adopt equity-sensitive interventions. Conditional cash transfers can help incentivize maternal and child health service uptake, while the expansion of community-based health insurance could reduce financial barriers that disproportionately affect poorer households [26]. Strengthening the role of community health workers to provide targeted outreach in underserved regions would further ensure that essential services reach those most at risk [27].

Embedding equity-adjusted planning into the work of ministries of health can help shift resource allocation from aggregate mortality alone toward inequality-driven burden. This would allow immunization, nutrition, and other essential health programs to be geographically weighted toward high-burden quintiles or districts, ensuring that investments reach the children who need them most [28]. This aligns with the aspirations of the African Union’s Agenda 2063 for inclusive development and is fundamental to achieving Universal Health Coverage (UHC). The approach extends beyond individual countries, as inequality patterns frequently cross borders. Regional bodies such as the African Union, ECOWAS, and SADC could play a coordinating role by developing harmonized, equity-focused strategies and accountability frameworks. Regular monitoring using tools such as HEAT would create the basis for shared regional benchmarks and progress tracking [19].

At the global level, financing mechanisms often emphasize overall burden of disease and cost-effectiveness, yet this framing risks overlooking the critical role of equity. Aligning donor frameworks with inequality-sensitive metrics could lead to more transformative outcomes [29]. Instruments such as Gavi’s equity accelerator or adaptations to the Global Fund allocation model offer entry points for channeling resources toward populations most affected by disparities [30, 31]. Mali’s PAF of −44.7% illustrates the potential impact of such a shift, as nearly half of child deaths could be prevented if interventions were effectively delivered to the poorest quintiles.

These findings also reinforce the need to move beyond national averages in the monitoring of the Sustainable Development Goals. Incorporating stratified and inequality-attributable indicators into Global Burden of Disease estimates and UN IGME reporting would provide a more accurate picture of where progress is lagging and why.

### Strengths and limitations

This study leverages harmonized, pre-validated estimates from UN IGME accessed via WHO HEAT, ensuring methodological consistency across countries. The use of both simple and complex inequality measures provides a multidimensional perspective, while the inclusion of over 6.5 million live births enhances statistical power and generalizability. Through combining mortality gradients with attributable fractions, the analysis advances the operationalization of precision public health.

However, limitations must be acknowledged. The reliance on survey-based estimates introduces potential recall bias and sampling error, although UN IGME smoothing and HEAT validation mitigate these risks. Additionally, the analysis is restricted to wealth and sex as stratifiers; other dimensions such as maternal education, geographic location, and ethnicity known to strongly influence U5M were excluded due to data constraints. Furthermore, HEAT does not allow multivariable adjustment or causal inference, meaning the results should be interpreted as descriptive and indicative rather than causal. Furthermore, while we ensured regional diversity in country selection, the analysis was not designed to formally test or explain regional variations in inequality. Future research could employ multivariable or comparative case-study designs to explore the specific contextual, health system, and policy factors that drive the stark inequality gradients observed in countries like Mali and Togo. A further limitation concerns the cross-country comparability of the wealth index. As the index is a relative measure of household wealth within each country, the absolute level of deprivation or affluence of a given quintile (e.g., ‘poorest’) is not equivalent across nations. Therefore, observed differences in mortality for the ‘poorest’ groups may reflect variations in national poverty levels and economic inequality, in addition to the relative socioeconomic gradient within countries. This limits direct comparisons of the absolute material circumstances of quintiles between countries. And the cross-sectional focus on 2022 precludes analysis of temporal trends, limiting insights into whether inequality is widening or narrowing over time.

## Conclusion and future directions

This analysis demonstrates that economic inequality is a defining driver of under-five mortality in Africa, with up to 45% of deaths in some countries linked to socioeconomic disparities. Through applying standardized methods from the WHO Health Equity Assessment Toolkit, the study goes beyond documenting gradients to quantify the proportion of deaths attributable to inequity, revealing the extent to which survival disadvantages are concentrated among the poorest children.

The policy message is clear: reducing child mortality requires not only scaling interventions but ensuring that they reach those most excluded. Equity-sensitive strategies such as conditional cash transfers, community-based health insurance, and tailored maternal-child health services can substantially reduce preventable deaths if directed toward the most vulnerable households. Regional institutions, including the African Union and ECOWAS, have an opportunity to embed inequality metrics in collective health strategies, while global donors should align financing frameworks with equity measures to maximize impact.

Future work should broaden the lens beyond wealth and sex to incorporate stratifiers such as maternal education, geography, and ethnicity, and employ tools like geospatial analysis, equity-adjusted cost-effectiveness, and dynamic modeling to sharpen intervention targeting. Embedding routine inequality monitoring into national health information systems will be essential for guiding action and holding systems accountable. This replicable analytical approach provides a blueprint for embedding equity metrics into health planning and accountability frameworks, which is a prerequisite for achieving SDG 3.2 and advancing UHC.

## Supplementary Information

Supplementary Material 1.

## Data Availability

The datasets analyzed for this study are openly accessible via the WHO Health Equity Assessment Toolkit, specifically the ‘Under-five mortality rate’ indicator from the United Nations Inter-agency Group for Child Mortality Estimation (UN IGME) databaseat: https://whoequity-heat-1.share.connect.posit.cloud/#.

## Abbreviations

CI: Confidence interval
D: Absolute difference
HEAT: Health Equity Assessment Toolkit
LMICs: Low- and Middle-Income Countries
PAR: Population Attributable Risk
PAF: Population Attributable Fraction
R: Relative ratio
RII: Relative Index of Inequality
SDGs: Sustainable Development Goals
SII: Slope Index of Inequality
U5M: Under-Five Mortality
U5MR: Under-five mortality rate
UN IGME: United Nations Inter-agency Group for Child Mortality Estimation
WHO: World Health Organization
