# Supplementary material for: Quantifying the attributable burden of economic inequality on under-five mortality across ten African countries: a WHO HEAT-based analysis
Source: BMC Public Health. 2025 Dec 17;26:263. doi: 10.1186/s12889-025-26002-y (PMC12821987; doi:10.1186/s12889-025-26002-y)
Supplement: Supplementary file 1 — Supplementary Material 1. [file 12889_2025_26002_MOESM1_ESM.docx]

**Supplementary Table S1: Under-Five Mortality Rates (per 1,000 live births) and Population Sizes by Wealth Quintile and Sex Across Ten African Countries, 2022.**

| **Country** | **Subgroup** | **Under-5 Mortality (per 1,000) (95% CI)** | **Population** |  | **Country** | **Subgroup** | **Under-5 Mortality (per 1,000) (95% CI)** | **Population** |
| --- | --- | --- | --- | --- | --- | --- | --- | --- |
| **Liberia** | Quintile 1 (poorest) | 80.4 (56.4, 117.8) | 32,318 |  | **Niger** | Quintile 1 (poorest) | 123.7 (95.4, 160.5) | 222,598 |
|  | Quintile 2 | 80.1 (56.8, 115.3) | 32,324 |  |  | Quintile 2 | 141.1 (109.9, 180.1) | 222,593 |
|  | Quintile 3 | 75.1 (53.6, 107.4) | 32,319 |  |  | Quintile 3 | 127.3 (100.0, 161.2) | 222,596 |
|  | Quintile 4 | 67.6 (47.4, 98.3) | 32,325 |  |  | Quintile 4 | 111.8 (86.1, 143.6) | 222,593 |
|  | Quintile 5 (richest) | 62.8 (43.5, 92.5) | 32,322 |  |  | Quintile 5 (richest) | 82.8 (62.5, 109.3) | 222,590 |
|  | Sex: Female | 67.2 (48.4, 96.2) | 79,300 |  |  | Sex: Female | 113.8 (90.4, 143.9) | 545,204 |
|  | Sex: Male | 79.0 (56.7, 112.8) | 82,282 |  |  | Sex: Male | 120.5 (95.6, 152.0) | 568,636 |
| **Madagascar** | Quintile 1 (poorest) | 79.6 (63.1, 101.1) | 177,440 |  | **Rwanda** | Quintile 1 (poorest) | 48.1 (27.8, 84.4) | 80,148 |
|  | Quintile 2 | 76.3 (60.6, 95.5) | 177,432 |  |  | Quintile 2 | 41.7 (24.2, 73.2) | 80,145 |
|  | Quintile 3 | 70.0 (56.5, 87.0) | 177,440 |  |  | Quintile 3 | 38.0 (22.3, 66.1) | 80,144 |
|  | Quintile 4 | 59.8 (47.3, 75.7) | 177,430 |  |  | Quintile 4 | 34.4 (19.9, 60.4) | 80,132 |
|  | Quintile 5 (richest) | 43.1 (33.4, 55.3) | 177,447 |  |  | Quintile 5 (richest) | 27.9 (15.9, 49.7) | 80,146 |
|  | Sex: Female | 60.3 (48.9, 74.4) | 435,730 |  |  | Sex: Female | 34.6 (20.3, 59.9) | 198,055 |
|  | Sex: Male | 70.9 (57.8, 87.4) | 452,539 |  |  | Sex: Male | 41.3 (24.3, 72.0) | 203,165 |
| **Malawi** | Quintile 1 (poorest) | 47.0 (27.7, 79.0) | 130,072 |  | **South Sudan** | Quintile 1 (poorest) | 106.2 (33.5, 260.8) | 62,594 |
|  | Quintile 2 | 45.7 (27.0, 76.3) | 130,072 |  |  | Quintile 2 | 107.6 (34.7, 260.7) | 62,594 |
|  | Quintile 3 | 40.9 (24.2, 68.2) | 130,062 |  |  | Quintile 3 | 99.3 (32.2, 239.3) | 62,595 |
|  | Quintile 4 | 36.4 (21.4, 61.4) | 130,068 |  |  | Quintile 4 | 85.9 (27.9, 211.0) | 62,592 |
|  | Quintile 5 (richest) | 30.5 (17.9, 51.7) | 130,088 |  |  | Quintile 5 (richest) | 94.9 (29.9, 233.6) | 62,597 |
|  | Sex: Female | 35.4 (21.2, 59.0) | 324,111 |  |  | Sex: Female | 93.9 (30.6, 226.5) | 153,771 |
|  | Sex: Male | 44.4 (26.5, 74.0) | 328,414 |  |  | Sex: Male | 103.7 (33.3, 248.9) | 158,884 |
| **Mali** | Quintile 1 (poorest) | 118.9 (90.1, 156.2) | 179,543 |  | **Togo** | Quintile 1 (poorest) | 80.1 (57.1, 110.9) | 54,643 |
|  | Quintile 2 | 111.1 (84.8, 145.4) | 179,546 |  |  | Quintile 2 | 72.1 (51.6, 100.0) | 54,642 |
|  | Quintile 3 | 101.6 (78.6, 131.7) | 179,542 |  |  | Quintile 3 | 63.5 (45.8, 86.5) | 54,634 |
|  | Quintile 4 | 85.2 (64.4, 112.4) | 179,542 |  |  | Quintile 4 | 51.5 (36.4, 71.3) | 54,646 |
|  | Quintile 5 (richest) | 51.9 (38.3, 69.7) | 179,542 |  |  | Quintile 5 (richest) | 34.7 (24.1, 49.2) | 54,639 |
|  | Sex: Female | 88.4 (68.6, 114.8) | 442,049 |  |  | Sex: Female | 55.3 (40.2, 75.4) | 134,948 |
|  | Sex: Male | 98.6 (76.6, 127.8) | 456,917 |  |  | Sex: Male | 65.1 (47.3, 88.6) | 138,600 |
| **Mozambique** | Quintile 1 (poorest) | 78.0 (49.0, 126.5) | 232,218 |  | **Uganda** | Quintile 1 (poorest) | 50.8 (33.8, 75.8) | 334,191 |
|  | Quintile 2 | 73.8 (46.6, 119.2) | 232,207 |  |  | Quintile 2 | 44.6 (29.5, 66.3) | 334,187 |
|  | Quintile 3 | 68.0 (43.2, 109.1) | 232,215 |  |  | Quintile 3 | 40.1 (27.0, 59.0) | 334,206 |
|  | Quintile 4 | 60.6 (38.2, 98.4) | 232,209 |  |  | Quintile 4 | 38.2 (25.4, 56.6) | 334,186 |
|  | Quintile 5 (richest) | 50.7 (31.5, 82.4) | 232,218 |  |  | Quintile 5 (richest) | 29.0 (19.0, 43.6) | 334,181 |
|  | Sex: Female | 61.8 (39.3, 98.5) | 574,588 |  |  | Sex: Female | 36.1 (24.4, 53.0) | 825,259 |
|  | Sex: Male | 70.6 (45.1, 112.4) | 585,943 |  |  | Sex: Male | 44.7 (30.3, 65.6) | 848,271 |
